# ﻿Contribution to the knowledge of the genus *Calcyopa* Stüning, 2000 (Lepidoptera, Geometridae, Ennominae, Boarmiini), with description of a new species

**DOI:** 10.3897/zookeys.1233.142955

**Published:** 2025-03-28

**Authors:** Bo Liu

**Affiliations:** 1 Coconut Research Institute, Chinese Academy of Tropical Agricultural Sciences, Wenchang, Hainan 571339, China Coconut Research Institute, Chinese Academy of Tropical Agricultural Sciences Wenchang China

**Keywords:** *Calcyopahainana*, DNA barcode, identification key, new species, species groups, taxonomic history

## Abstract

The genus *Calcyopa* Stüning, 2000, is briefly reviewed. A new species, *Calcyopahainana* Liu, **sp. nov.**, is described from Hainan Province, China. Within the genus *Calcyopa*, two species groups are identified, characterized by shared traits yet distinguished by a set of consistent features. The *difoveata*-group, comprises *C.difoveata*, *C.fansipana* and *C.hainana***sp. nov.**, and the *rosearia*-group, includes *C.rosearia*, *C.prasina* and *C.subprasina*. The relationship of both species groups is discussed, and an identification key of all known *Calcyopa* species is presented. Illustrations are provided for adult males and females of the *difoveata*-group, along with their genitalia, except for *C.fansipana*, which is known only from males. DNA barcodes are provided for the type species and the newly described species.

## ﻿Introduction

The genus-group name *Calichodes* was first published by Warren (1897: 246) with the description of *Calichodesfoveata* from Penang (Peninsular Malaysia). However, Warren did not mention it as a new genus, as the author usually did. Therefore, Wehrli (1943: 544) considered the name to be merely a manuscript name (“Warren M. S.”) and proposed it for a new subgenus of *Boarmia*, with a different type species, B. (Calichodes) difoveata Wehrli. Subsequently, Fletcher (1979: 32) only referenced *Calichodes* Wehrli, 1943, and raised it to the rank of a genus. Holloway ([1994]: 251) redefined *Calichodes* Warren as a monotypic genus, distinct from *Calichodes* Wehrli, and emphasized, based on a personal communication with I. W. B. Nye, that “the earlier usage is valid under the International Code of Zoological Nomenclature and therefore has priority”. Parsons et al. (1999: 100) followed this treatment and mentioned only *Calichodes* Warren, 1897, but included the type species *difoveata* of *Calichodes* Wehrli, without addressing the later invalid usage of Wehrli.

Consequently, the generic name *Calcyopa* was proposed by Stüning (2000: 134) as a replacement name for *Calichodes* Wehrli, 1943, with Boarmia (Calichodes) difoveata Wehrli, 1943 designated as the type species. Additionally, *Calcyopaprasina* Stüning, 2000 was described from Thailand, with its distribution extending from Vietnam, Peninsular Malaysia, and Myanmar to northern India and Nepal. Furthermore, the latter author included two other species, *Ectropisrosearia* Joannis, 1929 from “Tonkin” (N. Vietnam), Yunnan (China), Thailand, and Sumatra (Indonesia), and an undescribed species from Vietnam in this genus. Two decades later, two new species – *Calcyopafansipana* Sato, 2022 from N. Vietnam and *Calcyopasubprasina* Sato, 2022 from Laos – were added to the genus. Rajaei et al. (2022) mistakenly considered *Calcyopa* Stüning as a replacement name for *Calichodes* Warren and included all the species mentioned under *Calichodes* Warren in the catalogue of Parsons et al. (1999) under *Calcyopa* Stüning, 2000.

Currently, five species of the genus *Calcyopa* are known, and this paper describes a new species from Hainan Island, China.

## ﻿Material and methods

### ﻿Specimen collection

The study is based on moth specimens housed in the following collections: Coconut Research Institute, Chinese Academy of Tropical Agricultural Sciences, Wenchang, China (CRICATAS); Institute of Zoology, Chinese Academy of Sciences, Beijing, China (IZCAS); Natural History Museum, London, United Kingdom (NHMUK); Institute for Agro-Environmental Sciences, NARO, Tsukuba, Japan (NIAES); Zoologisches Forschungsmuseum Alexander Koenig, Bonn, Germany (ZFMK); Zoologische Staatssammlung München, Germany (ZSM).

### ﻿Morphology

Terminology of the wing venation follows the Comstock-Needham System (Comstock, 1918) as adopted for Geometridae by Scoble (1992) and Hausmann (2001), while genitalia terminology is based on Klots (1970) and Skou and Sihvonen (2015). For genitalia examination, abdomens were removed and placed in a hot 10% KOH solution. Genitalia were then dissected in 10% ethanol and stained with Chlorazol Black E. Photographs of adult moths were taken using a Nikon camera (D750) equipped with a Nikon lens (AF-S Micro 60 mm f/2.8G ED). Photos of genitalia were taken with a digital camera (KUY NICE E31SPM) attached to a Nikon microscope (SMZ745T). Focus-stacked images were generated using Helicon Focus software (version 8.2.2 Pro).

### ﻿DNA barcoding

Genomic DNA was extracted from the legs of dried adult specimens, and the barcode fragments were amplified using primers pairs: LCO-1490 and HCO-2198 or LepF1 and LepR1 (Folmer et al. 1994; Hebert et al. 2004). The obtained sequences (658 bp) were deposited in the Barcode of Life Data Systems (BOLD: Ratnasingham and Hebert 2007; http://www.boldsystems.org). All sequences mentioned in this study were obtained from the BOLD Systems. Genetic distances between species are reported as uncorrected pairwise distances (p-distance).

## ﻿Taxonomic account

### ﻿ Calcyopa

Taxon classificationAnimaliaLepidopteraGeometridae

Stüning, 2000

4DDD0DBB-2F75-57CE-90A6-6A78C707FC9A

Calcyopa Stüning, 2000, *Moths of Nepal, Part 6*. Tinea Vol. 16 (Suppl. 1):134. Type species: Boarmia (Calichodes) difoveata Wehrli, 1943.

#### Generic characters.

**Figure 1.**
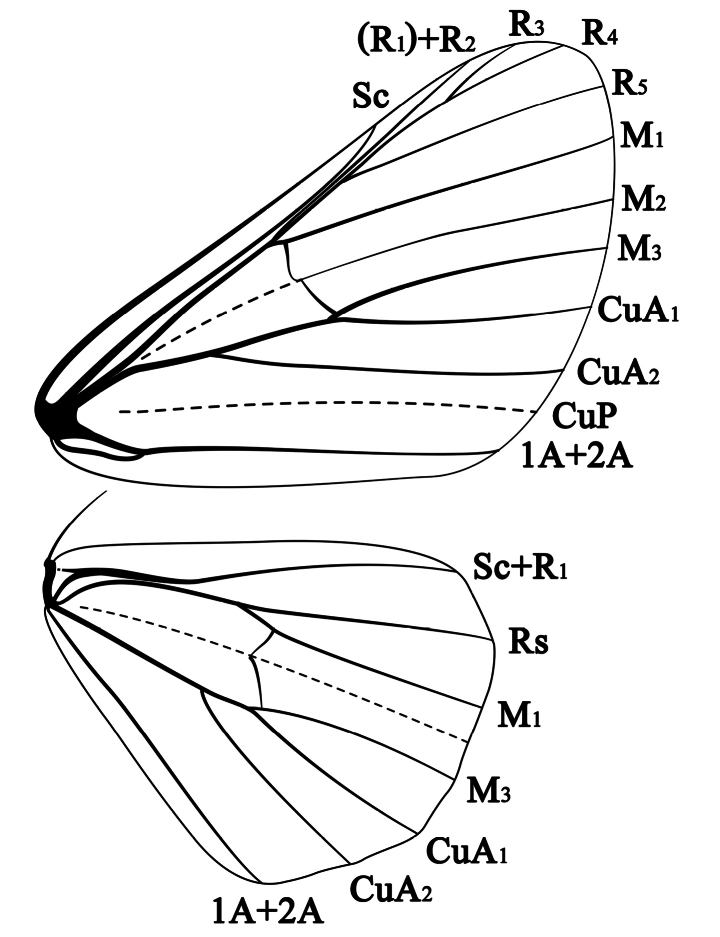
Wing venation of *Calcyopadifoveata*.

Small ennomine geometrid moths with forewing length 11–14 mm. Ground color light to dark grey or brown, with distinct black antemedial and postmedial lines on both wings. ***Head*.** Male antennae fasciculate, with two pairs of shortly conical, sclerotized, ciliate projections latero-ventrally on each segment. Cilia curved ventrad, 3–4 times longer (depending on species) than diameter of flagellomeres. Female antennae with cilia much shorter, the sclerotized projections absent. Vertex covered with lamellar scales. Frons smooth-scaled, not protruding. Labial palpi slightly extending beyond frons, basally with long scales. Proboscis rather short. Chaetosemata present, near eye margin. ***Thorax*.** Patagia and tegulae densely covered with somewhat longer lamellar scales, distal end of tegulae with a few long hair-scales. Legs slender, index of spurs 0-2-4, hind tibia not dilated, without scent brush (= hair-pencil of authors) in males. Forewings moderately elongate, apex angled, termen smoothly curved, with a double fovea in males. Antemedial and postmedial lines conspicuous on both wings. Postmedial line on forewing curved outward between M_1_ and M_3_, with the extent of curvature varying among species; outside postmedial line between veins M_3_ and CuA_1_ or even CuA_2_ with a dark, round, oval or squarish patch (absent in *C.rosearia* only) and a faint, square pale patch outside the strongly dentate submarginal line (absent in *C.rosearia*). Marginal line consisting of small, black dots in the middle between veins, alternating with groups of dark fringe-scales. Hindwing with apex rounded, termen minutely concave between vein-ends, marginal dots rather lunulate. Tornus without distinct spot (elongate black spot present near tornus in *C.prasina* and *C.subprasina*) Postmedial line straighter than on forewing. Discal dot distinct, visible on both wings, but larger on forewing. Underside with pattern paler. ***Venation*** (Fig. 1). R_1_ and R_2_ coincident (distal branch of R_1_ reduced, only R_2_ reaching costa closer to apex), the base of the combined veins running closely parallel to vein Sc and the stem of R_3-5_. (R_1_) +R_2_ and the stalk of R_3-5_ both arising from the same position, shortly before anterior angle of cell. M_2_ from the middle of the discoidal vein. CuA_1_ from shortly before posterior angle of cell. Hindwing: Sc+R_1_ running closely parallel to upper vein of cell for a short distance; Rs arising widely before anterior angle of cell; M_2_ absent, represented by a fold; CuA_1_ from shortly before posterior angle of cell; 3A absent. Folds through cells of both wings and those replacing CuP in forewings and M_2_ in hindwings very vague. ***Pregenital abdomen*.** Tympanal organs of moderate size, without lacinia. Sterno-tympanal process and setal comb on third sternite absent. Lateral coremata on the posterior portion of the third segment only present in *C.difoveata* and *C.hainana* (very small and easily brushed off during abdominal preparation). Seventh segment strongly modified, short, sternite membranous, with a pair of external corematous lobes, both laterally with a dense row of fine setae; anterior invaginated coremata, with intersegmental opening between segments 6 and 7 absent (not “weak, scarcely invaginated” as described in Stüning (2000) for *C.difoveata*) in all *difoveata*-group species (present in *rosearia*-group species).

***Male genitalia*.** Uncus deeply divided, stout, triangular, apically straight, slightly curved ventrad in species of the *difoveata*-group (elongate, more deeply divided, distally slender, apical part strongly bent ventrad in *rosearia*-group species). Gnathos with broad, flattened lateral arms, central part strong, thorn-shaped, elongate, slightly curved, pointed in *difoveata*-group species (central part delicate, spine-shaped in *rosearia*-group species). Juxta small, basally a rounded plate, extended into a narrow arm distally, slightly cup-shaped and laterally bifurcate at apex. Valvae acutely triangular. Basal costal arm free, standing obliquely upright towards the upper valva margin, apically dilated, with long bristles. Saccular process stick-like, spined at apex only (*C.difoveata*) or without spines (*C.hainana* and *C.fansipana*) (spined over half or more of its length in *rosearia*-group species). Aedeagus short and stout, apically with a long, acutely triangular, sclerotized process, shaft distally with short spines or groups of strong teeth, vesica with a small row of cornuti or a round diverticulum covered with numerous spicules in *difoveata*-group species (aedeagus smaller, narrower, without distal extension and without teeth on shaft and vesica without cornuti in *rosearia*-group species).

***Female genitalia*.** Ovipositor short, papillae anales rounded, scarcely setose. Needle-like sclerotization between their bases present. Anterior apophyses short, about ½ length of posterior apophyses. Lamella postvaginalis a rounded, rather small plate in *difoveata*-group species (a large, sclerotized plate with a wide rounded incision anteriorly in *rosearia*-group species). Introitus small, sclerotized; posterior part of bursa sclerotized, scobinate inside; anterior part of bursa swollen, broader than posterior part, signum consisting of 6 to 8 chains of small dentate projections in *difoveata*-group species (bursa short, rounded, signum built of dentate projections arranged to small chains or distributed over larger areas of the bursa surface in *rosearia*-group species).

#### Diagnosis.

The genus *Calcyopa* currently comprises a total of six species, including the newly described species presented in this study. These species can be distinctly divided into two groups, mainly based on the genitalia (both male and female) characters and the abdominal coremata. Despite their differences, all *Calcyopa* species share a deeply divided uncus, similarly triangular-shaped valvae, a free basal costal arm that is strongly setose at the apex, and a unique stick-like saccular process with or without spines in the male genitalia. These characters collectively set them apart from other related genera such as *Paracalcyopa* Sato, *Myrioblephara* Warren, and *Chrysoblephara* Holloway. The closely related genus *Necyopa* Walker exhibits most of these traits; however, its saccular process is replaced by a strong, longitudinal fold that extends close to the apex of the valva. In the present study, we separate all the known *Calcyopa* species into two groups: the *C.difoveata* species group, which includes *C.difoveata*, *C.fansipana* and *C.hainana*; and the *C.rosearia* species group, which comprises *C.rosearia*, *C.prasina* and *C.subprasina*. The main diagnostic characters of these two species groups are outlined in Table 1. While the two species groups share several common characters, they are also distinguished by a set of stable features. For the time being, we tentatively retain all of these species within the genus *Calcyopa*. However, depending on further molecular evidence, there might be a possibility of establishing a new genus for the *C.rosearia* species group or merging it with the genus *Necyopa* Walker.

**Table 1.** Diagnostic characters separating the *Calcyopadifoveata* and *C.rosearia* species groups.

Diagnostic characters	*C.difoveata* species group	*C.rosearia* species group
Intersegmental abdominal coremata between ST 6 and ST 7	absent	present
Uncus	moderately long, apex slightly curved ventrad	rather long, apex strongly bent ventrad
Saccular process	without spines or spined at apex only	spined over half its length
Aedeagus	rather stout, apical sclerotization long, tapering; cornuti spine-like	thin, apical sclerotization absent or indistinct, cornuti absent
Lamella postvaginalis	rather small, rounded	large, with a large, round incision medio-anteriorly
Introitus	small, sclerotized	large, membranous
Corpus bursa	long, cylindrical, proximally slightly inflated	short, oval or squarish

### ﻿ Calcyopa difoveata

Taxon classificationAnimaliaLepidopteraGeometridae

(Wehrli, 1943)

B921389F-F17E-519E-BD33-D48299A16A9C

**Figures 2–13.**
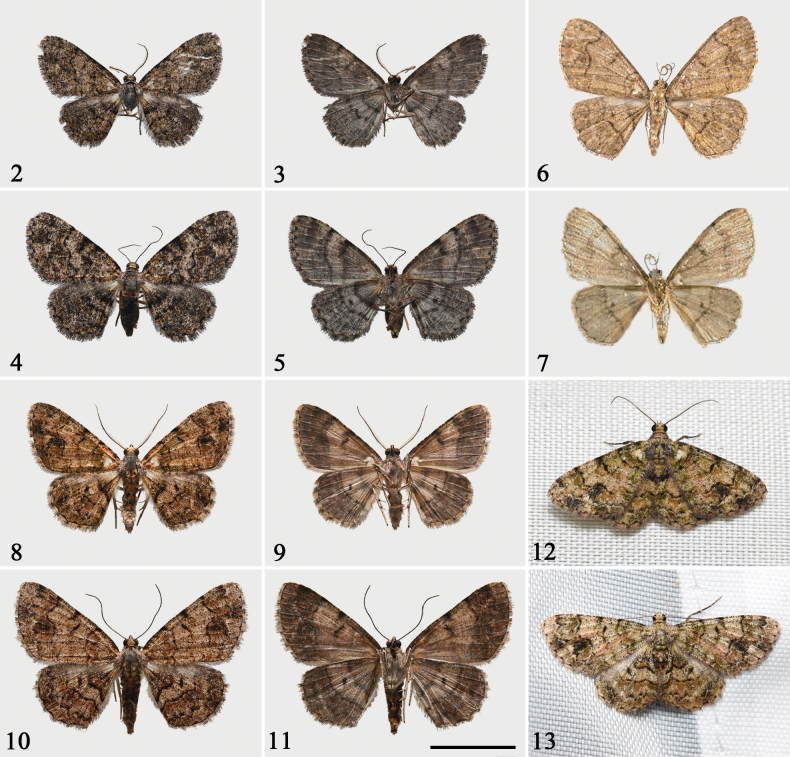
Adults of *Calcyopa* species. **2–5***C.difoveata*, Hainan, China, CRICATAS**2** male, upperside **3** ditto, underside **4** female, upperside **5** ditto, underside **6***C.fansipana*, holotype, male, upperside, N. Vietnam, NIAES**7** ditto, underside **8–11***C.hainana* sp. nov., Hainan, China, CRICATAS/ IZCAS**8** holotype, male, upperside **9** ditto, underside **10** paratype, female, upperside **11** ditto, underside **12, 13** specimens of *C.hainana* sp. nov. in resting position, type series, Hainan, China **12** male **13** female. Scale bar: 1 cm.

**Figures 14–19.**
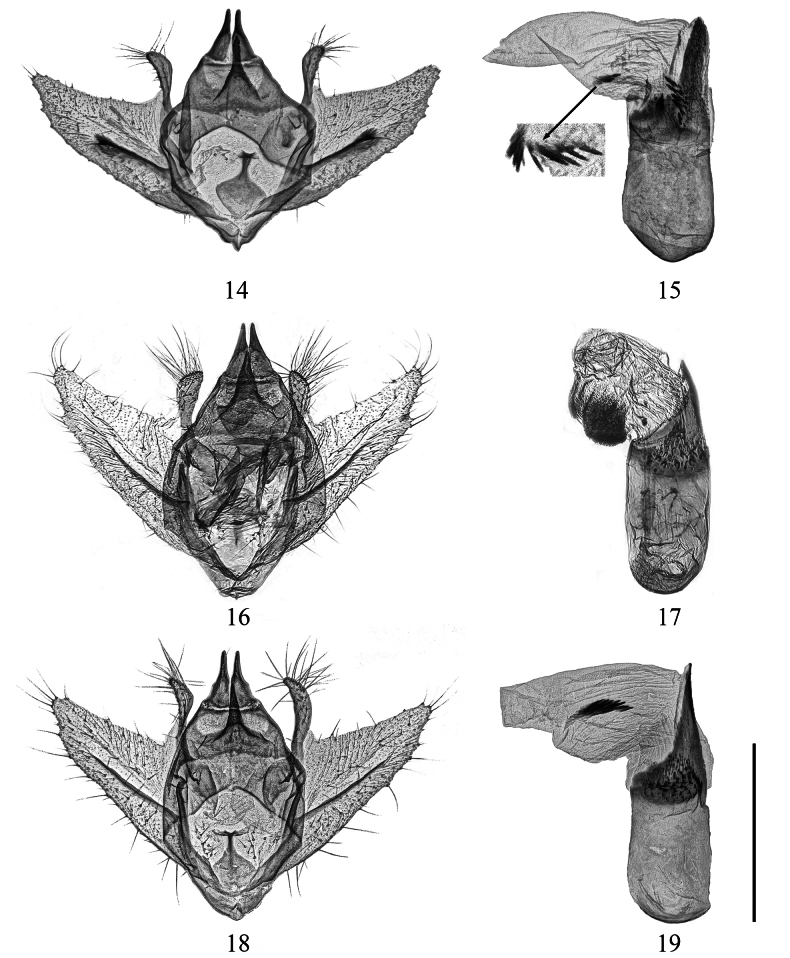
Male genitalia of *Calcyopa* species. **14, 15***C.difoveata*, Hainan, China **14** genitalia capsule **15** aedeagus, close-up of cornuti at the bottom left **16, 17***C.fansipana*, holotype, N. Vietnam, gen. prep. no. RS8731 **16** genitalia capsule **17** aedeagus **18, 19***C.hainana* sp. nov., paratype, Hainan, China, gen. prep. no. CRICATAS00101 **18** genitalia capsule **19** aedeagus. Scale bar: 1 mm.

**Figures 20, 21.**
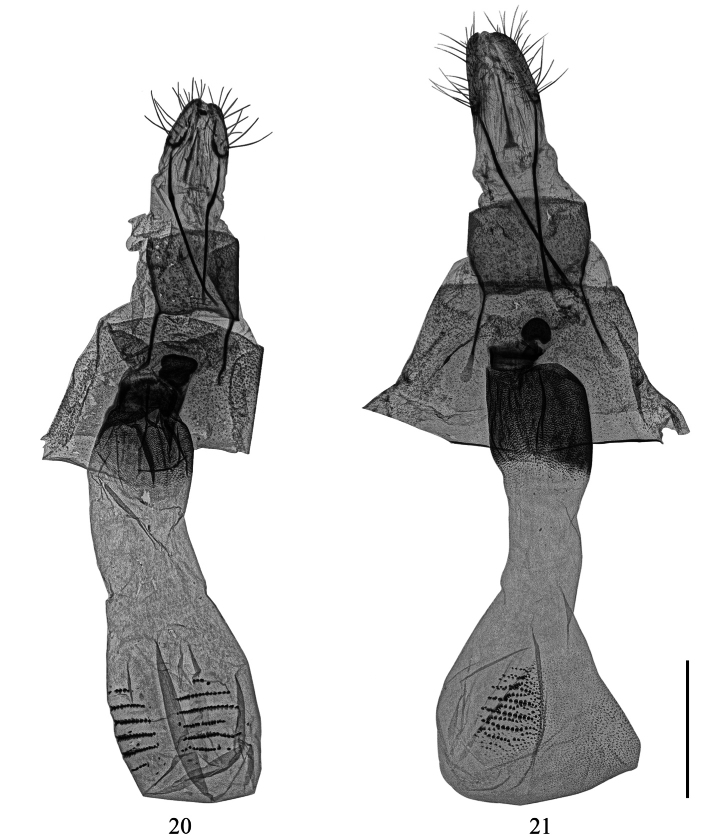
Female genitalia of *Calcyopa* species. **20***C.difoveata*, Hainan, China **21***C.hainana* sp. nov., paratype, Hainan, China, gen. prep. no. CRICATAS00103. Scale bar: 1 mm.

Figs 2–5 , 14 , 15 , 20

Boarmia (Calichodes) difoveata Wehrli, 1943, in: Seitz, Gross-Schmett. Erde 4 (Suppl.): 544. Type-locality: “West-Tien-Mu-Shan, Chekiang” (West Tianmushan, Zhejiang Province, China)
Calichodes difoveata Wehrli: Fletcher, 1979, In: Nye IWB (ed.), The Generic Names of Moths of the World 3: 32; Parsons et al. 1999, in: Scoble, Geometrid Moths of the World, A Catalogue, 1: 100.
Aethalura lushanalis Sato, 1987, Japan Heterocerists’ J. 144: 289, 290, figs 3, 4, 8. Synonymized with C.difoveata by Stüning (2000).
Calcyopa difoveata : Stüning, 2000, Tinea 16 (Suppl. 1): 135, fig. 1507; Sato 2020, in: Kishida Y, Moths of Laos, Part 1, Tinea 25 (Suppl. 2): 81, pl. 28, fig. 30; Sato 2022, Tinea 26 (3): 228, 230, figs 9, 10, 24, 36.

#### Type material examined.

***Lectotype***: China – **Zhejiang Province** • ♂; Pz. Chekiang, West-tien-mu-shan (= Zhejiang, West Tianmushan); 1600 m; 26 Apr. 1932; H. Höne leg.; gen. prep. slide no. 2451-DS; ZFMK (des. Stüning, 2000).

***Paralectotypes***: China – **Zhejiang Province** • 2 ♂♂, 1 ♀; same locality and collector as lectotype; 30 Apr. 1932; ZFMK. – **Guangdong Province** • 1 ♀; Lienping (Heyuan City, Lianping County); May; H. Höne leg.; coll. Wehrli, ZFMK. **Remark**: Wehrli (1943: 544) mentioned seven syntypes that were collected between 23 April and 13 June. Two female paralectotypes not included in the preceding material examined are not in coll. ZFMK, but should be the two specimens given to coll. NHMUK in exchange (1961).

#### Additional material examined.

China – **Zhejiang Province** • 4 ♂♂, 2 ♀♀; same locality and collector as lectotype; 3 Apr., 4 Apr., 10 Apr., 17 Apr. (2), 18 Apr. 1932; ZFMK • 5 ♂♂, 2 ♀♀; same locality and collector as lectotype; 400 m; mid-April 1936; gen. glycerol no. 6/B1♂; ZFMK • 1 ♀; Chekiang, Wenchow (= Zhejiang, Wenzhou); 18 Apr. 1939; H. Höne leg.; ZFMK • 3 ♂♂, 1 ♀; same locality and collector as for preceding; June 1939; ZFMK • 1 ♂, 2 ♀♀; same locality and collector as for preceding; July 1939; gen. glycerol no. 6/B3♀; ZFMK. – **Fujian Province** • 1 ♂; Fukien, Kuatun (= Fujian, Guadun); 27.40°N, 117.40°E; 2300 m; 3 Apr. 1938; J. Klapperich leg.; ZFMK. – **Guangdong Province** • 2 ♂♂, 3 ♀♀; Canton or Lienping; ZFMK. – **Taiwan Province** • 2 ♂♂ (paratypes of *Aethaluralushanalis* Sato, 1987); Nantou Hsien, Lushan-wenchuan; 13–14 Aug. 1983; R. Sato leg.; ZFMK • 2 ♀♀; Kaohsiung, Liukuei Sanping; 650 m; 24–26 Jul. 1987; R. Sato leg.; ZFMK • 1 ♂; Nantou Co., Hueisun Forest; 600 m; 22 Jun. 1993; F. Aulombard & J. Plante leg.; ZFMK • 1 ♂, 1 ♀; same locality and collectors as for preceding; 570–800 m; 28/29 Sep. 1992; ZFMK. – **Hainan Province** • 1 ♂, 1 ♀; Wuzhishan; 1333 m; 10 Jan. 2008; V. Siniaev leg.; ZSM • 5 ♂♂, 3 ♀♀; Wuzhishan; 756 m; 25 Mar. 2023; Bo Liu leg.; CATASCRI.

#### Diagnosis.

The diagnostic characters are given under the newly described species.

#### Distribution.

China (Zhejiang, Fujian, Jiangxi, Guangdong, Hainan, Taiwan), Vietnam, Laos.

#### Genetic data.

The Barcode Index Number for *Calcyopadifoveata* is BOLD: AAH0723 (*N* = 3, Sample IDs: CRICATAS00104, BC ZSM Lep 16015, BC ZSM Lep 15995).

#### Remarks.

This species is now recorded for the first time from Hainan Island, China. Some specimens from Hainan show minor differences in genitalia compared to those from Taiwan and Vietnam, including variation in the thickness of the gnathos in male genitalia as well as the shape of the lamella postvaginalis in female genitalia. At present, we treat these as infrasubspecific variations.

### ﻿ Calcyopa fansipana

Taxon classificationAnimaliaLepidopteraGeometridae

Sato, 2022

021361F1-B418-59D0-8BAD-1F753A2DB818

Figs 6 , 7 , 16 , 17

Calcyopa fansipana Sato, 2022, Tinea 26 (3): 226, figs 1, 2, 23. Type-locality: Mt Fan-si-pan, N. Vietnam.

#### Type material examined.

***Holotype***: Vietnam • ♂; N. Vietnam, Cha-pa (Sa Pa), Mt Fan-si-pan, N. Seite; 22°17'N, 103°44'E; 1600 m; 21–23 Apr. 1995; leg. Sinjaev & Sammler, ex coll. A. Schintlmeister; Prim Urwald; NIAES.

***Paratype***: Vietnam • 1 ♂; same data as holotype; NIAES.

#### Additional material examined.

Vietnam • 5 ♂♂; same locality and elevation as holotype; 01–05 Mar. 1995; ex coll./ leg. Dr. R. Brechlin; ZFMK • 1 ♂; same locality and collector as for preceding; 1520 m; ZFMK.

#### Diagnosis.

Diagnostic characters are given under the newly described species. The male adult and genitalia are illustrated in Sato (2022), but we depict them here again for comparison with the other two species of *difoveata*-group. The female is still unknown at present.

#### Distribution.

Vietnam.

#### Genetic data.

No data available.

#### Remarks.

Stüning (2000: 135) already mentioned this species as “an undescribed species also from Vietnam”, which was later described as *fansipana* by Sato (2022: 226). He wrote in the description: “Third abdominal sternite with setal comb” and “Hind tibia with hair-pencil”. These two characters were mentioned by an unexplainable error (Sato 2023, pers. comm.). Both setal comb and hair-pencil are absent in all species of the genus *Calcyopa*, which, of course, was known to Sato.

### ﻿ Calcyopa hainana sp. nov.

Taxon classificationAnimaliaLepidopteraGeometridae

46EF748F-026C-59FF-B0CA-F8D4ECF2CB83

https://zoobank.org/8AC4C54D-B7E3-4F34-B811-E5AB81F959CF

Figs 8–13 , 18 , 19 , 21

#### Type material.

***Holotype***: China – **Hainan Province** • ♂; Lingshui, Diaoluoshan; 922 m; 16–19 Apr. 2024; Bo Liu & Wei Yan leg.; CRICATAS/ IZCAS.

***Paratypes***: (12 ♂♂, 21 ♀♀) China – **Hainan Province** • 1 ♂; Wuzhishan; 1333 m; V. Siniaev leg.; 10 Jan. 2008; ZSM • 2 ♂♂, 2 ♀♀; same locality as holotype; 20 Apr. 2023; Bo Liu leg.; gen. prep. nos. CRICATAS00101, CRICATAS00103; CRICATAS/ IZCAS • 4 ♀♀; same locality and collectors as holotype; 05–07 Mar. 2024; CRICATAS/ IZCAS • 1 ♂, 1 ♀; same locality as holotype; 01–03 Apr. 2024; Bo Liu, Wei Lin & Miaofeng Xu leg.; CRICATAS/ IZCAS • 4 ♂♂, 5 ♀♀; same data as holotype; CRICATAS/ IZCAS • 4 ♂♂, 9 ♀♀; same locality and collectors as holotype; 07–12 May 2024; CRICATAS/ IZCAS/ ZFMK.

#### Diagnosis.

This species closely resembles *C.difoveata* and, though much less, also *C.fansipana*, but can be distinguished by several morphological characters: The postmedial line of the forewing between veins M_1_ and M_3_ exhibits a more pronounced outward curve than in *C.difoveata*, similar to that in *C.fansipana*. Moreover, the light-brown ground color of the upperside and the absence of broad, dark marginal bands on the underside of the latter are also diagnostic. The free costal arm of the valva is longer and narrower in *C.hainana* than in the other two congeners and the saccular process lacks spines. The triangular apex of the aedeagus is more pointed and elongated compared to *C.difoveata* and *C.fansipana*. Additionally, the cornuti in *C.hainana* are composed of a group of long juxtaposed spines, while in *C.difoveata* they consist of shorter spines, and in *C.fansipana* they form a dense cluster of minute spines covering a round diverticulum of the vesica. A row of rather large spines and a big single tooth present apically on the shaft of aedeagus of *C.difoveata* are replaced by a large number of smaller denticles in *C.hainana*.

#### Description.

Forewing length 11.1–11.9 mm in males, 11.7–14.3 mm in females. Ground color brownish-green in fresh specimens, but gradually fading to brown over time, with numerous scattered dark scales and distinct dark antemedial and postmedial lines on both wings. ***Head*.** Male antennae fasciculate, with two pairs of shortly conical, sclerotized, ciliate projections latero-ventrally on each segment. Female antennae with cilia much shorter, and sclerotized projections absent. The longest cilia are about 3 times the diameter of the flagellum segments in males and about 0.5 times the diameter of the flagellum segments in females. Vertex covered with lamellar, pale brown scales. Frons smooth-scaled, not protruding. Labial palpi slightly extending beyond frons, basally with long, dark scales, third segment small. Proboscis rather short. Chaetosemata present, near eye margin. ***Thorax*.** Patagia and tegulae densely covered with somewhat longer lamellar scales, distal end of tegulae with a few long hair-scales. Wings brownish, scattered with numerous dark scales. Forewings moderately elongate, apex angled, termen smoothly curved, with a double fovea in males. Antemedial line fine, visible. Discal spot small, streak-like. Postmedial line outcurved between veins M_1_ and M_3_, at CuA_2_, and at 1A+2A. Outside postmedial line between M_2_ and CuA_1_ with a round black patch anteriorly and a square pale patch posteriorly. Subterminal line zigzag, white, with a dark inner edge. Terminal line black, with a series of dark spots between the veins. Hindwings with apex rounded, termen minutely concave between vein-ends. Basal area densely covered with numerous black scales. Antemedial line broad, outcurved at lower cell vein and inner margin. Discal spot small, streak-like. Postmedial line outcurved between veins M_1_ and M_3_, and at inner margin. Subterminal zigzag, white, with a dark edge inside. Terminal line black, with dark spots between the veins. Underside blurry and paler, with a broad, dark band outside postmedial line on both wings. Antemedial line faint on forewing, but clearly visible on hindwing. Postmedial line barely outcurved on both wings. Legs slender, index of spurs 0-2-4, hind tibia not dilated, without scent brush in males. ***Pregenital abdomen*.** Tympanal organs, sterno-tympanal process, setal comb, and abdominal coremata as mentioned in the generic description. Abdomen laterally with several pairs of scale brushes on segments 2, 3, 4, 5, 6 and 7.

***Male genitalia*.** Uncus deeply divided, triangular, apical part straight, slightly curved ventrad. Gnathos with broad, flattened lateral arms, central part strong, thorn-shaped, elongate, slightly curved. Valvae acutely triangular. Basal costal arm free, standing obliquely upright towards the upper valva margin, apically dilated, with long bristles. Saccular process stick-like, without spines. Juxta small, basally a rounded plate, extended into a narrow arm distally, slightly cup-shaped and laterally bifurcate at apex. Aedeagus short and stout, apically with a long, acutely triangular, sclerotized process; shaft distally with a group of small serrate projections; vesica with a small row of long-spined cornuti. Bulbus ejaculatorius long, about four times as long as the aedeagus shaft.

***Female genitalia*.** Ovipositor short, papillae anales scarcely setose. Anterior apophyses short, about ½ length of posterior apophyses. Ventral longitudinal sclerotization needle-like, basally dilated, slightly triangular. Lamella postvaginalis small, round or oval, bottom concave at center. Introitus bursae small, sclerotized, significantly narrower than the posterior part of bursa, setting into a small sternite pocket. Posterior part of bursa slightly longer than anterior part, posterior one-third sclerotized, scobinate inside. Anterior part of bursa swollen, triangular, noticeably broader than posterior part, consisting of 7 to 8 chains of small serrate projections on both opposed inner sides.

#### Etymology.

The specific name of “*hainana*” is derived from the type locality, Hainan Island, China.

#### Distribution.

China (Hainan).

#### Genetic data.

The Barcode Index Number for *Calcyopahainana* is BOLD: AAH2362 (*N* = 2, Sample IDs: CRICATAS00101, BC ZSM Lep 16053). The genetic distance of *C.hainana* from *C.difoveata* (*N* = 3, Sample IDs: CRICATAS00104, BC ZSM Lep 16015, BC ZSM Lep 15995) ranges from 5.32% to 5.62% (p-distance).

### ﻿Key to all known *Calcyopa* species, based on male and female genitalia

**Table:** 

1	Uncus stout, deeply bifid until half its length, moderately long, apically slightly curved ventrad; saccular process without spines or spined at apex only; aedeagus large, rather stout, apical sclerotization long, tapering; cornuti spine-like; corpus bursae long, cylindrical, proximally slightly inflated; lamella postvaginalis rather small, rounded	**2 (*difoveata* -group)**
–	Uncus rather long and slender, deeply bifid until ¾ of its length, apically strongly bent ventrad; saccular process spined over half its length; aedeagus small, finer, apical sclerotization indistinct; cornuti absent; corpus bursae short, inflated, triangular or squarish; lamella postvaginalis large, rectangular, with a large, round incision medio-anteriorly	**4 (*rosearia* -group)**
2	Saccular process spined at the tip	***Calcyopadifoveata***
–	Saccular process less developed, without spines	**3**
3	Costal arm of valva slender; cornuti a group of long, juxtaposed spines	***C.hainana* sp. nov.**
–	Costal arm of valva stouter; cornuti smaller, arranged in two groups	***C.fansipana***
4	Signum a small group of dentate processes, partly arranged in short lines	***C.rosearia***
–	Signum consisting of two groups of spines or oblique grooves	**5**
5	Signum consisting of two groups of many minute spines, covering large areas of bursa	***C.subprasina***
–	Signum consisting of two oblique grooves covered with spines internally	***C.prasina***

## Supplementary Material

XML Treatment for Calcyopa

XML Treatment for Calcyopa difoveata

XML Treatment for Calcyopa fansipana

XML Treatment for Calcyopa hainana

## ﻿Additional information

### Author ORCID

Bo Liu https://orcid.org/0009-0008-7003-4659

### Data availability

All of the data that support the findings of this study are available in the main text.
